# Future Clinical Trials in DIPG: Bringing Epigenetics to the Clinic

**DOI:** 10.3389/fonc.2015.00148

**Published:** 2015-07-01

**Authors:** Andres Morales La Madrid, Rintaro Hashizume, Mark W. Kieran

**Affiliations:** ^1^Pediatric Neuro-Oncology, Department of Pediatric Hematology and Oncology, Hospital Sant Joan de Deu, Barcelona, Spain; ^2^Department of Neurological Surgery, Feinberg School of Medicine, Northwestern University, Chicago, IL, USA; ^3^Department of Biochemistry and Molecular Genetics, Feinberg School of Medicine, Northwestern University, Chicago, IL, USA; ^4^Pediatric Neuro-Oncology, Department of Pediatric Hematology/Oncology, Dana-Farber Cancer Institute, Boston Children’s Hospital, Boston, MA, USA

**Keywords:** DIPG, epigenetics, clinical trials, children

## Abstract

In spite of major recent advances in diffuse intrinsic pontine glioma (DIPG) molecular characterization, this body of knowledge has not yet translated into better treatments. To date, more than 250 clinical trials evaluating radiotherapy along with conventional cytotoxic chemotherapy as well as newer biologic agents have failed to improve the dismal outcome when compared to palliative radiation alone. The biology of DIPG remained unknown until recently when the neurosurgical expertise along with the recognition by the scientific and clinical community of the importance of tissue sampling at diagnosis; ideally, in the context of a clinical trial and by trained neurosurgical teams to maximize patient safety. These pre-treatment tumor samples, and others coming from tissue obtained post-mortem, have yielded new insights into DIPG molecular pathogenesis. We now know that DIPG comprises a heterogeneous disease with variable molecular phenotypes, different from adult high-grade glioma, other non-pontine pediatric high-grade gliomas, and even between pontine gliomas. The discovery of histone H3.3 or H3.1 mutations has been an important step forward in understanding tumor formation, maintenance, and progression. Pharmacologic reversal of DIPG histone demethylation therefore offers an important potential intervention strategy for the treatment of DIPG. To date, clinical trials of newly diagnosed or progressive DIPG with epigenetic (histone) modifiers have been unsuccessful. Whether this failure represents limited activity of the agents used, their CNS penetration, redundant pathways within the tumor, or the possibility that histone mutations are necessary only to initiate DIPGs but not maintain their growth, suggest that a great deal still needs to be elucidated in both the underlying biology of these pathways and the drugs designed to target them. In this review, we will discuss the role of both epigenetic and genetic mutations within DIPG and the development of treatment strategies directed against the unique abnormalities present in this disease.

## Introduction

Diffuse intrinsic pontine glioma (DIPG), the most frequent brainstem tumor in pediatrics, is one of the deadliest cancers among children. Until recently, diagnosis has been based on classic clinical and radiological presentation, with “no indication” for histologic confirmation in the vast majority of cases (1). This practice – with obvious absence of tumor tissue at diagnosis to study – has left DIPG basic and translational research behind other brain and non-CNS pediatric tumors in terms of their molecular characterization (2). Involved field radiation therapy of 54–60 Gy directed to the infiltrated pons, fractionated over a 6-week period, is the only palliative treatment that effectively, albeit temporarily, delays the progression of tumor growth, with improvement of most of the symptoms in approximately 70–80% of the cases. No survival benefit has been identified with alternative radiation strategies (i.e., hyper- or hypo-fractionation) versus conventional radiation. Similarly, radiation sensitization with different agents has been attempted without any improvement in outcome (1, 3). Unfortunately, the majority of children affected by DIPG will progress during the first year after diagnosis and only approximately 10% will survive past the second year. At tumor progression, no effective salvage therapy is available; although re-irradiation may provide transient benefit in a subset of patients (4). To date, more than 250 clinical trials including standard chemotherapeutic agents administered in different intensities and timings, biologic and targeted agents, immunotherapy, etc., along with radiation therapy have not significantly improved the event free survival (EFS) or overall survival (OS) of patients with DIPG (2). Not only have patients and their families not benefited from these therapies but also have suffered undesirable side effects of these futile interventions. Thus, with the increasing biologic data, we have now on DIPG, the scientific and clinical communities have the ability to start selecting more rationale treatment options that take into account the underlying pathways driving these tumors, the penetration of drugs into the tumor and the potential resistance pathways generated by the tumor (5).

## Recent Biologic Discoveries in DIPG

In the last decade, considerable efforts in revealing the cell of origin and underlying molecular make up of DIPG by different collaborative groups have yielded major discoveries at the genetic and epigenetic levels. Monje et al. proposed a Nestin^+^/Vimentin^+^ neural pontine precursor-like cell as a candidate for DIPG cell of origin, linked both anatomically – restricted to the ventral pons – and temporally to the incidence of DIPG. Additionally, the same group proposed that dysregulation of the Hedgehog pathway is of relevance in tumor development (6). At the molecular level, a considerable bulk of DIPG knowledge comes from biologic studies obtained from post-mortem series and also from the initiative of several groups, first by the French, in obtaining tumor tissue through stereotactic biopsies at diagnosis (6, 7). This procedure, in expert hands and in highly controlled circumstances, has shown to be safe with no mortality reported and limited associated morbidity (8–10). Of note, it must be emphasized that not all patients are appropriate to undergo a biopsy, not all neurosurgeons have the experience to perform these procedure, and not all institutions have the molecular pathology expertise or experience to obtain meaningful data from these very small, heterogeneous, and precious samples (1). In circumstances where biopsy is appropriate, deep molecular investigation of these samples – by whole genome sequencing, gene expression array, and DNA methylation array – have resulted in our ability to define DIPG in “biologic terms” rather than in merely radiologic or clinical ones (8).

Diffuse intrinsic pontine gliomas have a high frequency (around 80%) of mutually exclusive somatic mutations of *H3F3A* and *HIST1H3B* genes, resulting in replacement of lysine 27 by methionine (K27M) in the encoded histone H3.3 and H3.1. Interestingly, other pediatric midline high-grade gliomas share mutations in H3.3 but not the H3.1 mutations and cortical pediatric and adult malignant gliomas rarely share these mutations (11). The identification of these prevalent mutations in DIPG not only helps confirm the diagnosis at a molecular level but has also shown to provide relevant clinical and prognostic information in independent retrospective series. For example, H3.3 mutations are associated with shorted survival compared to H3.1 mutations. H3.1 mutated tumors tend to occur in younger patients and are often associated with other specific mutations (i.e., *ACVR1*) that are not observed in other midline or cortical malignant gliomas (8, 12–14). Other alterations that appear important in DIPG are mutations in *TP53* – identified in 60% of cases – *PI3K*, *EGFR*, *PTEN*,*ATRX*, and *PDGFRA* (7, 14–17). Thus, it is now clear that DIPG is different at the molecular level from other non-brainstem high-grade gliomas and other types of pediatric brain tumors. To go even further, we now appreciate that DIPG is not a single entity, but rather a complex and varied pathology comprised of separate molecularly defined sub-groups that share clinical and radiological features, as well as a grim prognosis. Moving forward, the development of new clinical trials should take into account the heterogeneity of DIPG, not only among different patients but also within different areas of the tumor of each patient, which may play a key role in tumor resistance to current therapies. The ability to detect histone mutations using immunohistochemical analysis of paraffin embedded formalin fixed tissue rather than more completed molecular sequencing should simplify our ability to identify these important patient subpopulations (18).

## Epigenetic Alterations in DIPG Gliomagenesis: “The Polycomb Connection”

**Figure 1:**
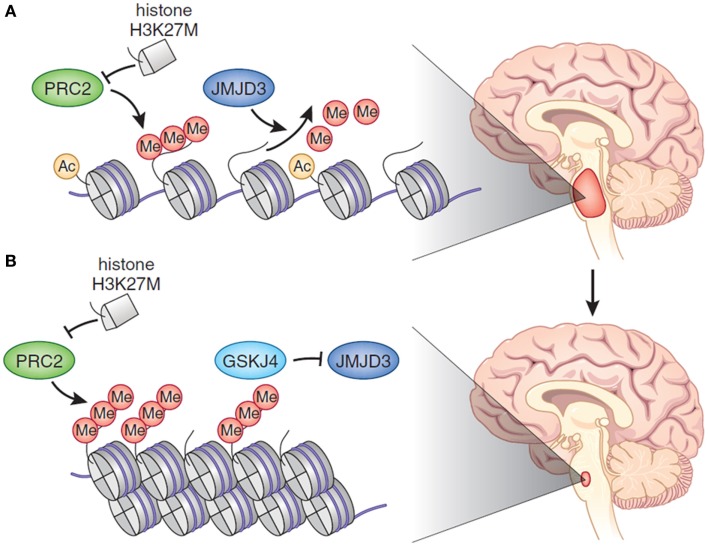
**(A)** In DIPG, there is global hypomethylation of Lys27 of H3, which promotes a more accessible chromatin state characterized by H3K27 acetylation and aberrant gene expression. Histone H3.1 or H3.3 harbors a K27M aberration. The mutant K27 histone inhibits PRC2, which is the major H3K27 methylase. **(B)** Treatment of DIPG with an epigenetic modifier – in the cartoon GSKJ4 – restores methylation at H3K27 toward the physiological state, causing tumor shrinkage. Reprinted by permission from Macmillan Publishers Ltd: Nature Genetics [May; 46(5):457–61], copyright 2014.

The role of epigenetic modifications in tumorigenesis, tumor maintenance, and progression has gained a lot of momentum in recent years, but has not yet been well established (19). Certainly, epigenetic changes, either alone or in combination with other mutations, may need to work in concert to drive cancer initiation, propagation, or both. This has led to a growing interest in identifying the specific role that epigenetic alterations play in DIPG and the potential effect of epigenetic modification as a way to treat and alter the natural history of this deadly cancer (11, 20). Recently, the mutation of the *H3F3A* gene has gained considerable attention for its potential role in tumorigenesis, and therefore, as a good candidate for targeted therapy. As mentioned above, approximately 80% of DIPG tumors contain mutations in genes that encode histones (H3.3 or H3.1), proteins that package DNA into chromatin. These mutations, which change lysine 27 to methionine (K27M), are believed to sequester polycomb repressive complex 2 (PRC2), which normally represses gene expression through histone methylation. When PRC2 is functionally inactivated, genes that should be silent are expressed, which is thought to drive cell transformation (Figure 1A). These alterations abolish a crucial site of regulatory post-translational methylation (PTM) (13, 21, 22). As such, tumor-derived histone gene mutations are thought to drive tumorigenesis in very specific cell lineage and developmental context by causing reduced histone K27 methylation and thereby altering gene expression in cells of the developing ventral pons. Interestingly, it is well known that the introduction of the H3.3K27M mutation into *p53*-null and nestin-expressing progenitors in the neonatal mouse brainstem is unable to generate gliomas, although it is sufficient to induce ectopic cell clusters in the mouse brain. This has led different groups to focus on the role that these epigenetic alterations play in DIPG initiation versus maintenance or proliferation (10, 12, 17, 18, 22). One model of DIPG was generated by differentiating human embryonic stem cells into neural progenitor cells and then transducing them with a viral vector carrying the gene encoding H3.3K27M. The expression of this gene was mitogenic only in neural progenitors derived from embryonic stem cells, and not in undifferentiated embryonic stem cells or astrocytes derived from these cells. This suggested that the histone mutation is oncogenic only in the appropriate cell type and cellular context. Importantly, only when progenitors also expressed an activated form of *PDGFRA* and lacked the *TP53* tumor suppressor could they give rise to gliomas after injection into the brainstem of mice. Moreover, even with all three genetic alterations present, the tumors grew slowly and lacked histological features of high-grade glioma (i.e., palisading necrosis and vascular proliferation) (23).

Recently, the effect of pharmacologic modulation of histone K27 methylation, by inhibiting H3K27 demethylation, has been demonstrated. The rational used was the following: by inhibiting the activity of JMJD3 H3K27 demethylase, K27 methylation would increase with a subsequent decrease in gene expression. The expected result was to inhibit cell proliferation in the K27M mutant DIPG cell lines derived from patient’s tumors. The use of the GSKJ4, an inhibitor of JMJD3 H3K27 demethylase in K27M-expressing cells revealed a dose-dependent inhibition of cellular viability, with 50% growth inhibition. GSKJ4 also led to more apoptosis of K27M-expressing cells and completely inhibited the clonal growth of all K27M-expressing cells. Similar results were obtained from *in vivo* experiment with the use of athymic (*nu*/*nu* genotype, BALB/c background) mice harboring brainstem K27M glioma xenografts. The drug showed significant reduction in the growth of K27M tumors engrafted in mouse brainstem and significantly extended animal survival with evidence of decreased proliferation and augmented apoptosis (Figure 1B). Drug showed good brain penetration including to the site of brainstem tumor development. According to the gene expression analysis, GSKJ4 treatment seemed to up-regulate the genes involving with apoptosis, cell cycle arrest, and cell differentiation, and down-regulate the genes involving with angiogenesis and cell proliferation (24, 25). It is of interest, and potential significance, that another recent study identified JMJD3 as a potential therapeutic target in treating T-cell acute lymphoblastic leukemia (T-ALL), in which JMJD3 expression is also elevated (26). Results from this study, in combination with results involving the inhibition of JMJD3 in DIPG, suggest that JMJD3 as an emerging therapeutic target in cancer treatment. More recently, Grasso et al. took a different approach integrating genomic and chemical screening data. By chemical screening using a DIPG culture panel, they identified panobinostat as one of the most active agents in the screen (27). Encouraging results in pre-clinical testing in cell lines and animal models of this FDA-approved potent HDAC inhibitor will likely take this drug to clinical trials soon. Interestingly, cell culture testing showed that there was synergy with the combination of panobinostat with GSKJ4 – and not with other tested drugs – depicting the relevance of identifying the appropriate drug combination for increasing the likelihood of clinical benefit (27).

## Future Directions and Implications in DIPG Clinical Trials Design

The discovery of K27M mutations in DIPG was an important step forward in understanding this tumor and promises to yield new approaches to treating the disease. However, several key aspects should be taken into account when designing future clinical trials for DIPG (Box 1). Historically, epigenetic modification (even before the discovery of the high prevalence of histone mutations in DIPG) with valproic acid, a drug that theoretically penetrates the blood–brain barrier, has been unsuccessful in most series (28). It is likely that this failure represents lack of effective drug levels in the CNS, off target activity, or alternative escaping pathways within the tumor. The COG ACNS0927 phase 1/2 clinical trial – currently closed to accrual – that tested suberoylanilide hydroxamic acid (SAHA, Vorinostat), a histone deacethylase inhibitor, and local irradiation, followed by maintenance SAHA in children with newly diagnosed DIPG, will show if this more potent epigenetic modifier has some impact on EFS and OS in DIPG. It is clear that we need to better understand what are the relevant and key pathways and how we can better target them (1, 2). The scientific community needs to continue working to create models that can be used to study DIPG biology and demonstrate that these models can be useful in identifying novel and more effective therapies. It seems that epigenetic regulation may play a key role in tumor formation and maintenance. Thus, it makes sense that drugs targeting these mechanisms will be ideal for testing in these models. However, these agents should meet the following requirements: they should inhibit the target, be delivered to the pons in effective concentrations with sufficient duration of exposure, and have an acceptable toxicity profile, taking into account the continuing normal development of pediatric patients with DIPG. Other considerations that need to be taken into account in the identification of optimal drug therapy include a detailed understanding of the target, when and where it is present within the tumor and on what other normal cell populations does it have important impact. Finally, understanding how best to measure clinical efficacy of novel agents using changes in the tumor on imaging, as well as the traditional measures of PFS and OS need to be determined. All these questions should be addressed before launching the next wave of clinical trials in order to decrease the likelihood of additional negative results (2). With the ongoing clinical trials, which include tumor biopsy at diagnosis, some of these questions will hopefully be answered. While a strong positive improvement in survival is optimal, it is critical that clinical trials that are negative have an ability to understand why the treatment did not work. Molecular profiles have proven to be prognostic and biomarkers for treatment options are now a real possibility. Selecting patients by specific genetic and epigenetic alterations as eligibility criteria for clinical trial enrollment needs to be considered. Unfortunately, the molecular advances made to date and identification of pathways mutated in these tumors has not resulted in improvement in either PFS or OS. One consideration that could address some of the disappointment in response to targeted therapy to date is re-biopsy at the time of progression, not just at autopsy as is now commonly performed. Tumor evolution and resistant clone selection are recognized phenomena in cancer biology and it is likely that these may play a role in DIPG treatment resistance and rapid tumor progression. Re-analysis of tumor tissue at progression may be needed to assess for the genetic and epigenetic changes over the pre-therapy sample, while also identifying “new” target engagement after exposure to a specific inhibitor or combination of inhibitors. With paired molecularly characterized tumor tissue (at diagnosis and after treatment) target modulation and engagement could be potentially more effectively achieved. If the scientific community decides to go down this path, the safety of the procedure will have to be analyzed first by teams with expertise in the field. The application of innovative approaches for upcoming clinical trials will be paramount in identifying more effective therapies. Importantly, combination phase I trials in oncology have shown to be feasible and safe (29). This strategy may decrease the time elapsing from drug development to drug testing in patients, increasing the number of drugs tested in a given period. Also, newer biologic targeted agents’ activity – including epigenetic modifiers – relies more on their “biologic effect,” rather than on its “dose effect,” with no need of escalating to the maximal tolerated dose (19). Therefore, the expected toxicity with each drug or combination of drugs is lower when compared with trials that include classic cytotoxic agents.

Box 1 **Key points in designing “epigenetic” future clinical trials for DIPG.**

- The identification of epigenetic dysregulation in DIPG pathogenesis opens new avenues in treating this disease.
- Prior “negative” clinical trials may represent lack of effective drug levels in the CNS, off target activity, or alternative escaping pathways within the tumor.
- Pre-clinical testing in representative cell cultures and appropriate animal models can be useful in identifying novel and more effective therapies.
- An ideal drug should be delivered in effective concentrations and inhibit the target, with sufficient duration of exposure, and have an acceptable toxicity profile.
- Early identification of responders by newer imaging techniques or biologic surrogates may avoid futile toxicities and unfulfilled hopes.
- Re-biopsy at time of progression should be considered in the near future (once safety issues are addressed), since the driving target identified in the tumor at diagnosis – and directing therapy – may be gone or altered at progression.
- Novel clinical trial design and combination of agents with different mechanism of action may offer a better chance for tumor control in this deadly entity.

Many questions remain to be answered in the understanding of this disease, including the cancer stem cell and appropriate context for DIPG initiation, as well as which mutations and epigenetic alterations contribute to tumor maintenance and progression. The high incidence and exclusivity of histone H3 mutations to DIPG highlights the importance of identifying the true cell of origin that is susceptible to these mutations, as well as how the signaling pathways cooperate to drive gliomagenesis in a specific developmental context. It is now well recognized that epigenetic changes influence many of the hallmarks of cancer, such as malignant self-renewal, differentiation blockade, evasion of cell death, and tissue invasiveness (11, 30). Thus, it is reasonable that future clinical trials should include epigenetic modifiers at least as part of the therapeutic schema. It is hoped that normal cells will not be significantly affected by epigenetic modifiers and thus may be well tolerated in pediatric patients. The “epigenetic vulnerability” of certain cancer cells in many ways mirrors the age old axiom of “oncogene addiction.” Some cancer cells are reliant on specific epigenetic pathways, whereas normal cells have alternative compensating pathways that protect them from these inhibitors (19). Further studies that address these and other questions will ultimately lead to more effective treatments for DIPG. Given the dismal prognosis associated with this disease, there will be strong incentive to move new treatments forward into clinical trials, but with a novel approach. It is likely that the key for more effective therapies will include “combination therapies.” It is likely that many of these new epigenetic drugs offer synergistic benefits, and may also synergize with other targeted agents. This “combination” approach will not only potentially increase therapeutic efficacy but also reduce the likelihood of drug resistance with higher chances for durable tumor control at presentation and hopefully one day result in a cure.

## Conflict of Interest Statement

The authors declare that the research was conducted in the absence of any commercial or financial relationships that could be construed as a potential conflict of interest.
